# FGF23 promotes prostate cancer progression

**DOI:** 10.18632/oncotarget.4174

**Published:** 2015-05-19

**Authors:** Shu Feng, Jianghua Wang, Yiqun Zhang, Chad J. Creighton, Michael Ittmann

**Affiliations:** ^1^ Department of Pathology and Immunology, Baylor College of Medicine and Michael E. DeBakey Dept. of Veterans Affairs Medical Center, Houston, Texas, USA; ^2^ Dan L. Duncan Cancer Center Division of Biostatistics, Baylor College of Medicine, Houston, Texas, USA; ^3^ Department of Medicine, Baylor College of Medicine, Houston, Texas, USA

**Keywords:** prostate cancer, FGF23, signal transduction, fibroblast growth factors, endocrine fibroblast growth factors

## Abstract

Prostate cancer is the most common cancer in US men and the second leading cause of cancer deaths. Fibroblast growth factor 23 (FGF23) is an endocrine FGF, normally expressed by osteocytes, which plays a critical role in phosphate homeostasis via a feedback loop involving the kidney and vitamin D. We now show that FGF23 is expressed as an autocrine growth factor in all prostate cancer cell lines tested and is present at increased levels in prostate cancer tissues. Exogenous FGF23 enhances proliferation, invasion and anchorage independent growth *in vitro* while FGF23 knockdown in prostate cancer cell lines decreases these phenotypes. FGF23 knockdown also decreases tumor growth *in vivo*. Given that classical FGFs and FGF19 are also increased in prostate cancer, we analyzed expression microarrays hybridized with RNAs from of LNCaP cells stimulated with FGF2, FGF19 or FGF23. The different FGF ligands induce overlapping as well as unique patterns of gene expression changes and thus are not redundant. We identified multiple genes whose expression is altered by FGF23 that are associated with prostate cancer initiation and progression. Thus FGF23 can potentially also act as an autocrine, paracrine and/or endocrine growth factor in prostate cancer that can promote prostate cancer progression.

## INTRODUCTION

Prostate cancer (PCa) is the most common visceral malignancy and the second leading cause of cancer deaths in men in the United States. There is compelling evidence from analysis of human tumor samples, *in vitro* studies and animal models that fibroblast growth receptors (FGFRs) are important in PCa initiation and progression [1-10]. The fibroblast growth factor (FGF) signaling network plays an important role in the development, tissue repair and tumorigenesis by regulating cell proliferation, migration, chemotaxis, morphogenesis and angiogenesis. Aberrant FGF signaling can promote tumor development by directly driving cancer cell proliferation, invasion and survival as well as by supporting tumor angiogenesis [1-10]. These observations make FGF signaling networks increasingly attractive as targets for therapeutic intervention in cancer.

Fibroblast growth factor 23 (FGF23) is a member of the endocrine FGF subfamily, which includes FGF19, FGF21 and FGF23. Endocrine FGFs are secreted into serum and they are stable in this environment, which allows them to act in an endocrine fashion. In addition they require Klotho (KL) or Klotho-β (KLB) as co-receptors for high affinity binding to FGF receptors (FGFR) in relevant target tissues. FGF23 signaling is mediated via the complex formed by FGF-23, FGFR (R1c, R3c or R4) and Klotho [11, 12]. We have previously shown that both FGFRs and KL are ubiquitously expressed in PCa [7, 8]. FGF23 is normally expressed in osteocytes and has a critical role in phosphate homeostasis as key component of an endocrine feedback loop between bone and the kidney, along with the vitamin D metabolite 1,25(OH)_2_D_3_ [13]. To date there is only limited evidence linking FGF23 to cancer, although it is well established that tumor induced osteomalacia is a result of FGF23 secretion by a number of tumor types, including prostate cancer [14]. Recently, three single nucleotide polymorphisms (SNPs) in the *FGF23 gene* were found to be associated with the development of prostate cancer [15]. In this study, we show that FGF23 can act as an endocrine, paracrine and/or aurocrine growth factor in PCa and plays an important role in PCa progression.

## RESULTS

### FGF23 is expressed in prostate cancer and prostate cancer cell lines

**Figure 1:**
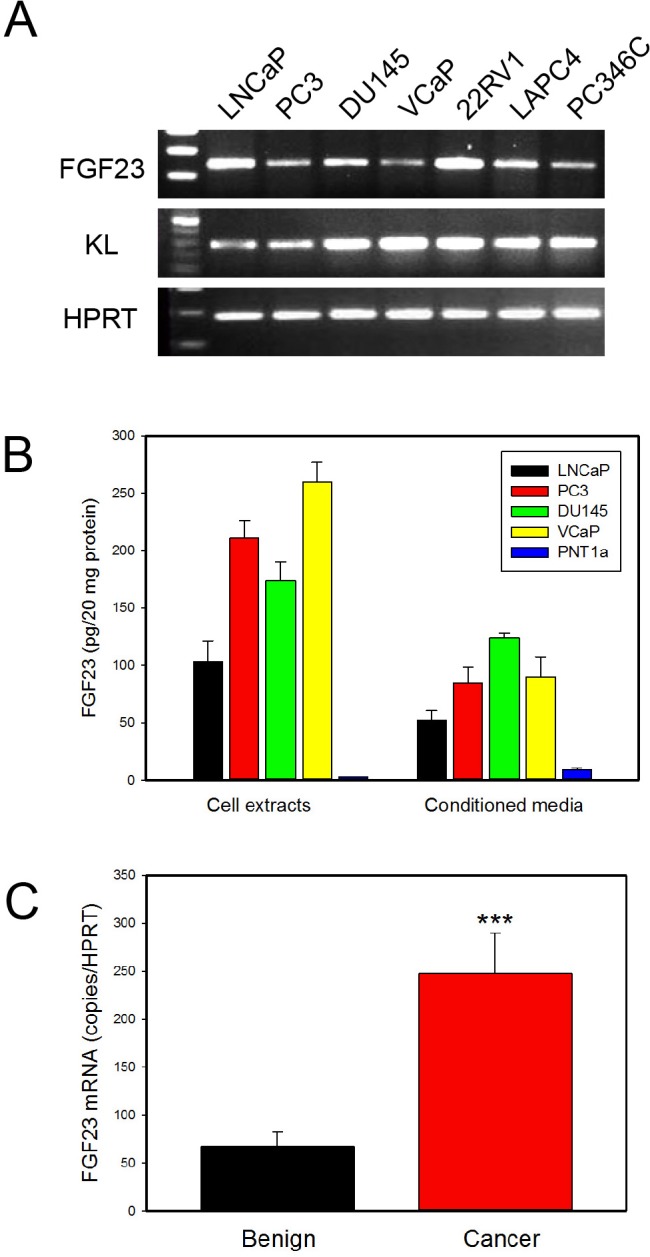
FGF23 is expressed in PCa cell lines and prostate and PCa tissues **A**. FGF23 and KL expression was determined by RT-PCR using RNAs from the indicated PCa cell lines. HPRT is a control. **B**. Expression of FGF23 in cell extracts and conditioned medium of PCa cell lines (LNCaP, PC3, DU145 and VCaP) and immortalized normal prostate epithelial cells (PNT1a). **C**. FGF23 mRNA expression in benign prostate and PCa tissues from radical prostatectomy specimens. Expression was normalized using HPRT mRNA. Mean +/− SEM is shown (n=36 benign; n=52 cancer). *** *p* < .001, t-test.

We initially screened PCa cell lines for expression of FGF23 by RT-PCR. All cell lines tested, including PC3, DU145, LNCaP, VCaP, 22RV1, LAPC4 and PC346C express detectable FGF23 mRNA as well as Klotho co-receptor (Fig. 1A). To quantitate FGF23 protein expression we carried out a FGF23 ELISA on cell extracts and conditioned media from the LNCaP, PC3, DU145 and VCaP PCa cell lines and PNT1a immortalized normal prostate epithelial cells. FGF23 protein was present in all the PCa cell lines in both cell extracts and conditioned media (Fig. 1B) but was barely detectable in PNT1a conditioned media (but not in cell extracts). Quantitative RT-PCR showed highest levels of FGF23 mRNA in LNCaP and lowest levels in PNT1a (Supplementary Fig. 1). Comparison with Fig. 1B shows that the mRNA levels were not directly proportional to protein levels, implying the possibility of post-transcriptional control of FGF23 protein levels. We also carried out Q-RT-PCR of RNAs from benign prostate and PCa tissues from radical prostatectomy specimens. FGF23 mRNA was detected in both benign and cancer tissues, with 3.6-fold higher levels in the cancer tissues (p<.001, t-test; Fig. 1C). We did not see a significant correlation of cancer FGF3 expression levels with clinical or pathological parameters, although the sample size is small, tempering this conclusion. Thus FGF23 is expressed in prostate cancer at increased levels.

### Exogenous FGF23 promotes prostate cancer cell proliferation, invasion and anchorage independent growth

**Figure 2:**
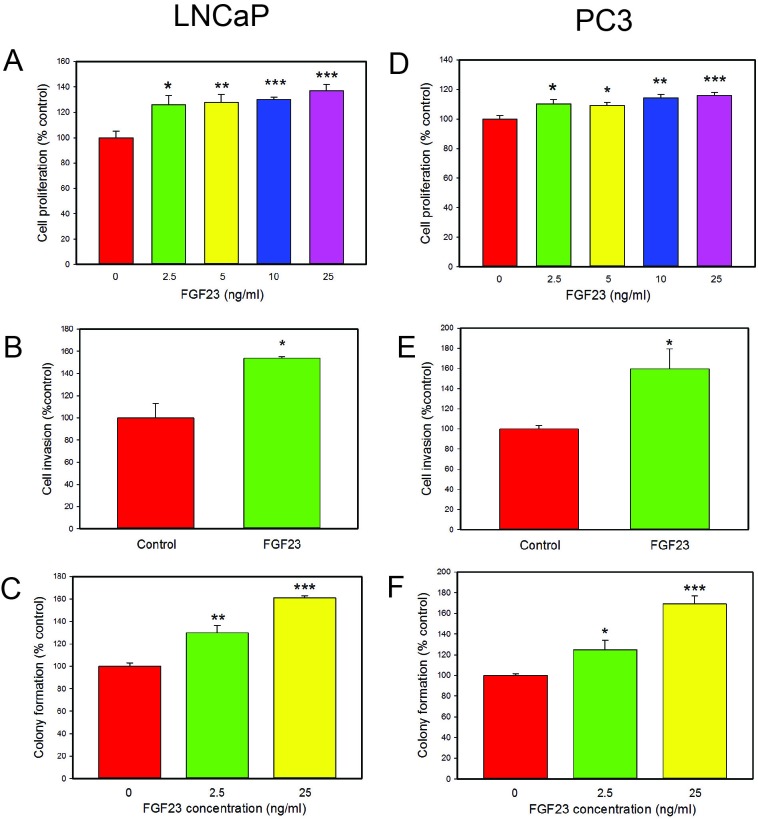
Biological effects of exogenous FGF23 on PCa cell lines. **A**, **D**. FGF23 induced proliferation. FGF23 was added to cultures of cells in serum free medium at the indicated concentration and cell number analyzed at 48 hrs. **B**, **E**. Invasion through Matrigel determined as described in Methods. **C**, **F**. Colony formation in soft agar. FGF23 or vehicle was added to cells in soft agar at the indicated concentration and colonies counted after 14 days. Data is expressed relative to vehicle only control (100%). Mean +/− standard error of the mean (SEM) for triplicates are shown for all experiments. Asterisks indicate statistically significant differences. * *p* <. 05; **<*p* < .01; *** *p* <. 001 by t-test.

**Figure 3:**
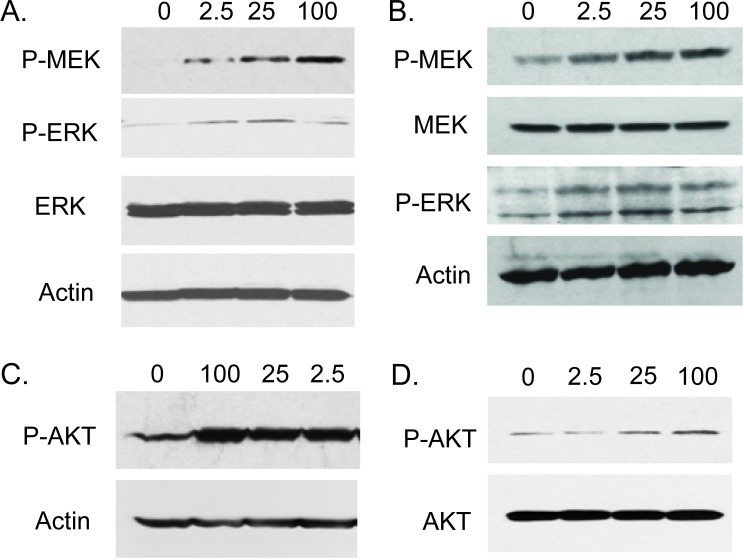
FGF23 activates MAP kinase and AKT pathways in PCa cells LNCaP (**A**, **C**) or PC3 (**B**, **D**) cells were serum starved for 24 hours and stimulated with the indicated concentration of FGF23 for 15 min in serum free medium. Cell lysates were prepared and Western blotting performed as described in Methods to assess activation of MAPK (A: LNCaP; B: PC3) and AKT signaling (C: LNCaP; D: PC3 ). β-actin is a loading control.

We next examined the impact of exogenous FGF23 on cellular phenotypes associated with cancer progression by adding exogenous FGF23 to LNCaP or PC3 PCa cells and measuring proliferation, invasion and soft agar colony formation relative to vehicle control treated cells. For LNCaP cells exogenous FGF23 increased proliferation by up to 37%, invasion by 54% and soft agar colony formation by up to 61% (Fig. 2A-2C). For PC3 cells exogenous FGF23 increased proliferation by up to 16% (modest, but statistically significant), invasion by 59% and soft agar colony formation by up to 69% (Fig. 2D-2F). Of note, FGF23 did not stimulate proliferation of PNT1a cells (data not shown). These findings indicate that exogenous FGF23 can alter tumor promoting phenotypes in PCa cells. Western blots of FGF23 stimulated PCa cells showed increased phosphorylation of both ERK and AKT in both cell lines, indicating activation of these pathways in PCa cells treated with FGF23 (Fig. 3).

### Knockdown of FGF23 inhibits tumor growth *in vivo*

**Figure 4:**
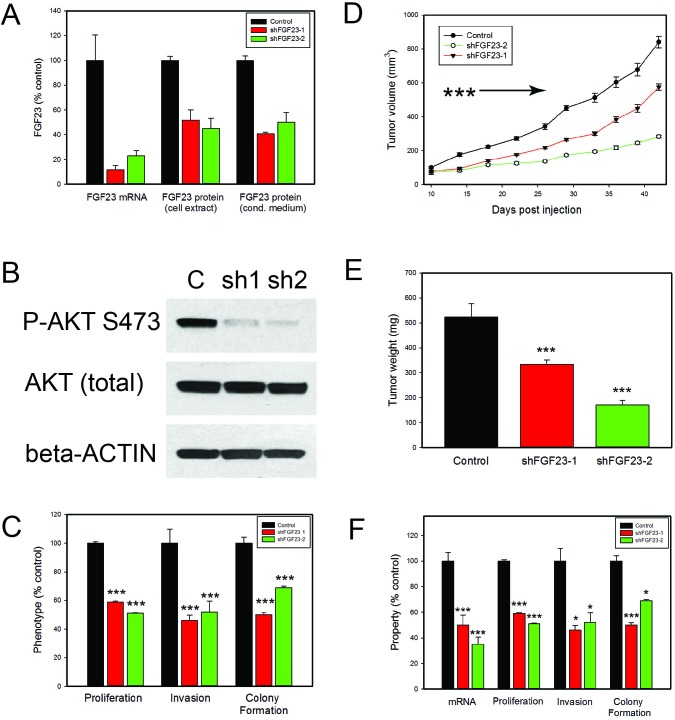
Biological effects of FGF23 knockdown on PCa cells **A**. FGF23 was stably knocked down using two different shRNAs and mRNA levels evaluated by Q-RT-PCR. FGF23 protein levels in cell extracts and conditioned medium were determined by ELISA. **B**. Western blot of PC3 knockdown cell lines and vector control with anti-phospho-AKT (Ser473) antibody. β-actin loading control is shown. **C**. Proliferation, invasion and soft agar colony formation in PC3 knockdown and vector control cell lines. Experimental data is expressed relative to vector control cells (100%). The mean +/−SEM is shown. **D**. PC3 cells with knockdown of FGF23 (FGF23 sH-1, n=30 and FGF23 sH-2, n=16) or vector controls (n=26) were inoculated subcutaneously on day 1. Calculated tumor volume is shown at intervals from day 10 to day 42. Mean +/− SEM is shown. Tumors from both knockdown cell lines were smaller than vector controls (p<.001, t-test ) from day 14 on as indicated by the arrow. **E**. Final tumor weights from xenograft experiment (mean +/− SEM). F. Proliferation, invasion and soft agar colony formation in LNCaP knockdown and vector control cell lines. Experimental data is expressed relative to vector control cells (100%). The mean +/−SEM is shown. Statistically significant differences from controls are indicated by asterisks for all experiments. * *p* <. 05; **<*p* <. 01; *** *p* <. 001 by t-test.

FGF 23 can enhance growth, invasion and anchorage independent growth and is expressed as an autocrine growth factor in all PCa cell lines tested. Both KL and FGFRs are ubiquitously expressed in PCa cells, suggesting a potential autocrine stimulatory loop. To determine the importance of autocrine FGF23 expression in the transformed phenotype we examined the impact of FGF23 knockdown with shRNAs on transformed phenotypes. Initial studies using transient transfection of FGF23 targeting shRNAs showed decreased FGF23 mRNA was associated with decreased cell numbers in PC3, LNCaP and 22RV1 cells at both 48 and 96 hours after tranfection in comparison to vector controls (data not shown). We therefore established stable knockdown cell lines using two independent FGF23 targeting shRNAs in PC3 cells. Stable knockdown of cell lines with knockdown of FGF23 mRNA and protein were successfully established in PC3 cells (Fig. 4A). Of note, mRNA knockdown (77 and 88%) was more effective than protein knockdown (50 to 59%) implying that there may some posttranscriptional mechanism to enhance FGF23 protein expression in the face of mRNA knockdown. FGF23 knockdown resulted in a marked decrease in AKT phosphorylation (S473) in PC3 cells as shown in Fig. 4B. *In vitro* studies with the knockdown cell lines showed significant decreases in proliferation (40-50%), invasion (50-55%) and anchorage independent growth (35-50%) relative to vector controls (Fig. 4C). To test the impact on tumor growth *in vivo* we carried out subcutaneous xenograft studies with knockdown and control cell lines in SCID mice. FGF23 knockdown resulted in significant decreases in tumor growth (Fig. 4D and 4E). We also established similar stable knockdown lines in LNCaP cells. This cell lines showed somewhat less potent knockdown (50 and 65% decreases in mRNA) but still showed significant decreases in proliferation, invasion and anchorage independent growth (Fig. 4F).

### FGF23 has both overlapping and unique downstream patterns of transcriptional activation and suppression compared to other FGFs

**Figure 5:**
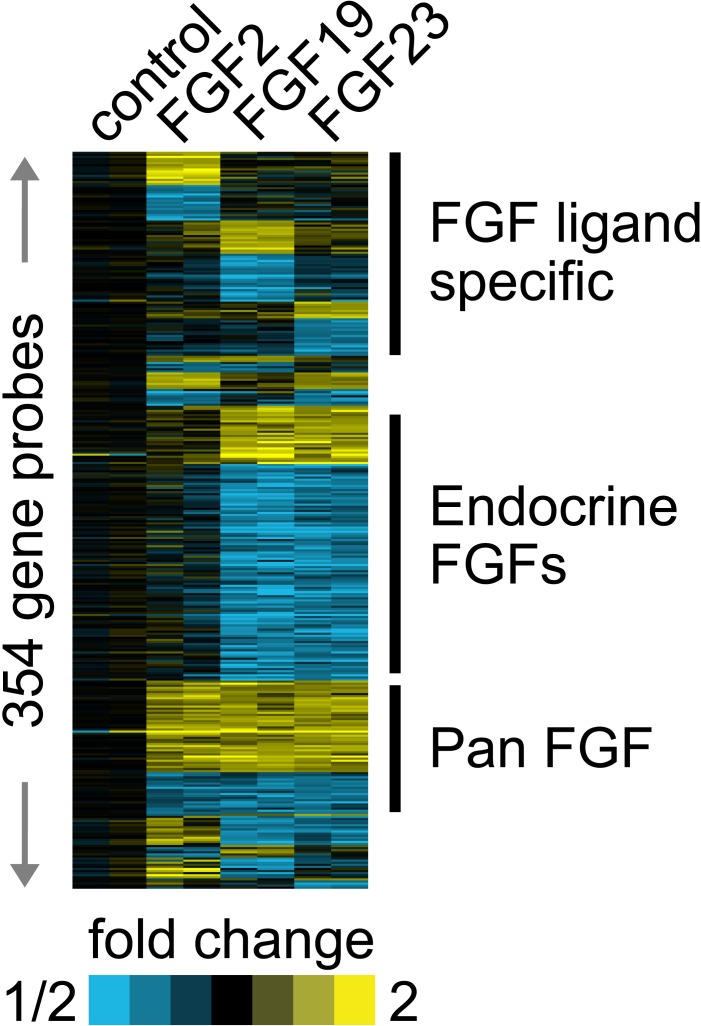
Different FGF ligands drive both common and unique gene expression changes Biological duplicates of LNCaP cells were placed in serum free medium for 24 hrs and then stimulated with FGF2, FGF19 or FGF23 for 24 hours. RNAs were collected, labeled and hybridized to Agilent 60K arrays. Genes with >1.4-fold or <0.7 fold decrease were indentified and a supervised cluster analysis performed. Yellow, up-regulation; blue, down-regulation.

**Figure 6:**
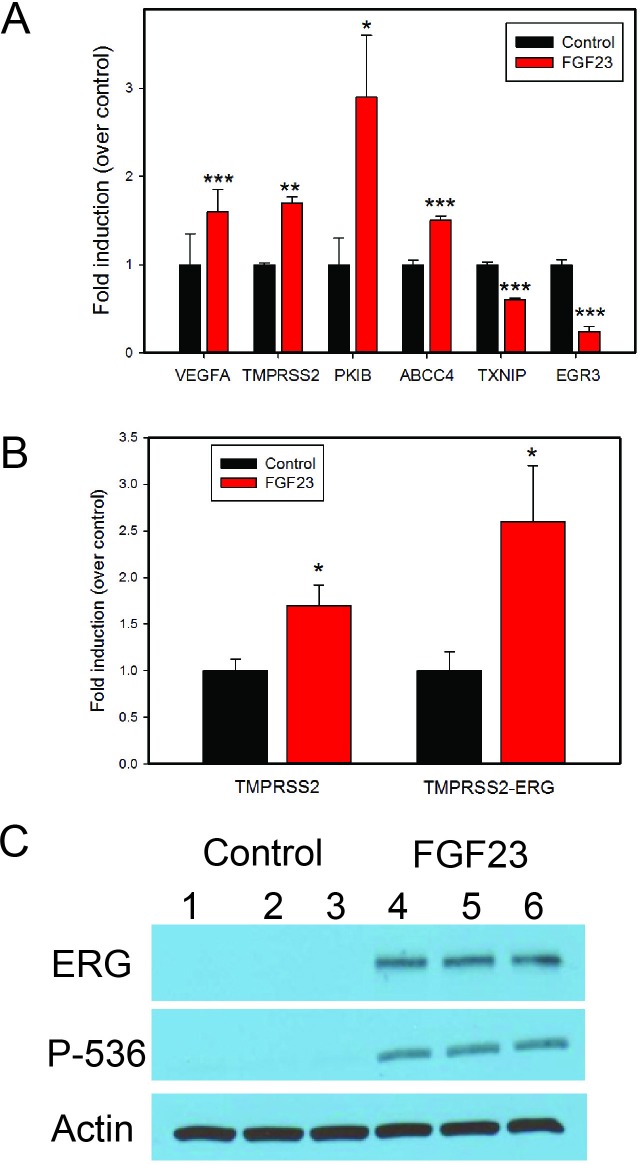
FGF23 alters expression of genes implicated in tumor progression **A**. Q-RT-PCR analysis of gene expression changes after FGF23 stimulation in LNCaP cells of cancer related genes identified in expression array analysis. **B**. Fold induction of TMPRSS2 and TMPRSS2/ERG fusion gene in VCAP cells after FGF23 stimulation in serum free medium without androgens. **C**. ERG protein levels in VCaP cells stimulated by FGF23 in serum free medium without androgens. Phospho-NF-kB p65-Ser536, which is increased by ERG in VCaP cells is also shown. β-actin is a loading control. Biological triplicates of vehicle treated and FGF23 treated cells are as indicated. * *p* < .05; **<*p* < .01; *** *p* < .001 by t-test.

Our group and others have shown increased expression of classical FGFs such FGF6 [2], FGF8 [16] and FGF17 [1] in PCa. We have also recently shown expression of FGF19, another endocrine FGF, in PCa [8]. This raises the question of whether different FGF ligands are activating the same transcriptional program in PCa cells or are there unique or partially overlapping activities as well? To begin to address this question we stimulated biological duplicates of LNCaP cells with FGF2 (classical FGF), FGF19 or FGF23 and collected mRNAs after 24 hrs. We then carried out expression array analysis using Agilent 60K expression arrays. A total of 354 probes showed fold changes of >1.4-fold up or <0.7-fold down in one or more treatments relative to vehicle control. We then carried a supervised cluster analysis of the expression data, which is shown in Fig. 5. It is clear that the different FGF ligands induce both unique and overlapping patterns of gene expression changes. For example, there are 3 blocks of genes with a total 98 probes that represent genes altered only by specific FGF ligands (Fig. 5, FGF ligand specific). On the other there is also a block of 64 genes which show the same pattern of regulation by all FGFs (Pan FGF genes). In addition, there is a large block of 132 genes that are altered only by the endocrine FGFs. The remaining 60 genes show all other potential combinations of up and/or downregulation by the three different FGF ligands. After removing redundant probes and noncoding genes this yielded a list of 271 unique protein encoding genes (Supplementary Table 1). Examination of the genes specifically upregulated by FGF2 reveals several genes that are known targets of classical FGFs including FOS [17], JUN [17], cyclin D2 [18] and ETV5 [19]. We then examined several genes of relevant to the transformed phenotype that were altered by FGF23 stimulation using Q-RT-PCR. VEGFA, PKIB and TMPRSS2 were increased by FGF23 stimulation in LNCaP cells while the tumor suppressor TXNIP was decreased (Fig. 6A). ABCC4, which is associated with chemotherapy resistance, was also increased. EGR3 expression, which is lower in aggressive prostate cancer, was also significantly decreased. Of note, Klotho expression was not affected. TMPRSS2 is an androgen regulated serine protease and is involved in the TMPRSS2/ERG gene fusion that occurs in 50% of PCas. We therefore examined the expression of both TMPRSS2 and the TMPRSS2/ERG in VCaP cells stimulated with FGF23. As shown in Fig. 6B there was a 1.7 and 2.5-fold increase in expression of these genes in response to FGF23 stimulation in the absence of androgens. The increased expression of the TMPRSS2/ERG fusion gene was confirmed by Western blotting (Fig. 6C). In addition, FGF23 increased phosphorylation of the NF-κB p65 subunit at its Ser536 site, which we have shown previously is downstream target of ERG [20] Thus FGF23 can alter expression of the TMPRSS2/ERG fusion gene and other genes involved in the neoplastic phenotype in PCa.

## DISCUSSION

FGF23 is a circulating endocrine FGF that plays a critical role in calcium and phosphate homeostasis. We now show that FGF23 is expressed as an autocrine growth factor expressed in all PCa cell lines tested that promotes the transformed phenotype, based on both *in vitro* and *in vivo* studies. In addition, exogenous FGF23 also promotes proliferation, invasion and soft agar colony formation *in vitro*. Thus FGF23 can potentially also act as an endocrine and/or paracrine growth factor in PCa. Endocrine FGFs are present in serum in healthy adults (approximate mean FGF23: 26 ng/l [21]). Furthermore, FGF23 is elevated in disease states, some of which have been tied to PCa risk and/or aggressiveness. Mean serum levels of FGF23 in patients with renal failure can be elevated 700 fold or more (~20,000 ng/l [21]). Patients with less severe renal dysfunction also have elevated FGF23 [22]. Of note, higher serum calcium, which is associated with higher FGF23 [21] is significantly associated with the incidence of fatal PCa [23, 24]. Finally, FGF23 is produced and secreted by osteocytes and thus can act as a paracrine factor for nearby tumor cells so that FGF23 is likely to be a significant source of FGF signaling in the bone microenvironment. Given that bone is by far the most common site of PCa metastasis, this may be clinically significant. Lee et al [25] have observed severe hypophosphatemia in a subgroup of patients with advanced PCa consistent with excess FGF23 activity and also report a bioinformatics analysis showing increased FGF23 mRNA expression in a subset of patients with metastatic PCa. These results complement our observations in localized PCa and the biological studies reported here. Thus FGF23 can play a role in PCa progression as an autocrine, paracrine and/or endocrine growth factor, the relative importance of each depending on tumor FGF23 expression level, tumor site and underlying disease states in the patient.

Prior studies in our laboratory and many others have shown that a number of classical FGFs are increased in PCa and we have recently shown that FGF19, an endocrine FGF, is expressed in PCa as well [8]. Of note, several PCa cell lines express both FGF19 and FGF23 (DU145, PC3, VCaP) while PNT1A cells express neither at significant levels. Our data shows that a classical FGF (FGF2) and the endocrine FGFs (FGF19 and FGF23) activate common, partially overlapping and unique patterns of gene expression, implying both common and unique activities of each ligand in PCa. Thus expression of these different FGF ligands is not redundant, despite the fact that they all signal through FGF receptors. Exactly how different genes are activated by different ligands, despite activation of the same receptors and at least some common pathways, such as ERK [7, 8], is not clear The Klotho co-receptor may influence downstream signaling and activate unique patterns of gene expression in conjunction with FGFRs accounting for the differences seen between FGF2 and the endocrine FGFs. Furthermore, the affinity of different FGF ligands for the four different FGFRs is not identical. However, the manner in which FGF ligands activate unique patterns of gene expression is still unclear and further studies are needed.

Based on our gene expression arrays we identified a number of downstream targets of FGF23 that are potentially relevant to enhancing the neoplastic phenotype and confirmed their altered expression by Q-RT-PCR. It should be noted that in some cases FGF2 and/or FGF19 can also alter expression of these genes so they are not all FGF23 specific (see Supplementary Table 1). VEGFA (Vascular endothelial growth factor A) belongs to a family of angiogenic growth actors. In addition to the paracrine role in endothelial cells to induce tumor neovascularization, autocrine effects of VEGFA in tumor progression and metastasis have been reported in PCa [26]. A consistent increase in VEGFA expression has been observed in primary tumor specimens as well as serum samples from prostate cancer patients [27]. PKIB(cAMP-dependent protein kinase inhibitor-b) is presumed to be one of the regulatory factors in the PKA pathway that can contribute to cancer cell viability and aggressive phenotype through functional linking between the PKA and Akt pathways [28] PKIB is overexpressed in aggressive castration-resistant PCa and its overexpression correlates with high Gleason score [28]. ABCC4 (human multidrug resistance-associated protein 4) is a member of a family of membrane transport proteins that may play an important role in cellular detoxification. It is an androgen-regulated gene that is increased in PCa [29]. It has been shown to impair response to docetaxel [30], an important chemotherapy agent for advanced PCa. Thus FGF23 can enhance expression of multiple genes associated with PCa progression or therapeutic resistance. However, we have not shown that these genes are actually critical for tumor progression *in vivo* in either in our PC3 subcutaneous model or LNCaP cells. Further experiments will be needed to systematically define the role of these FGF23 regulated genes in PCa progression.

TXNIP (Thioredoxin-interacting protein), is an endogenous inhibitor of the antioxidative function of thioredoxin and thus is an important regulator of redox homeostasis. TXNIP has been shown to be an important tumor suppressor, and its expression is dramatically reduced in various types of human tumors [31]. EGR3 (Early growth response 3) is a member of the early growth response family of transcription factors. EGR3 mRNA and protein are overexpressed in non-relapsing PCa but not in relapsing disease [32]. Thus FGF23 can also downregulate genes that demonstrate decreased expression in aggressive PCa.

The TMPRSS2 gene is androgen regulated and provides an androgen regulated promoter for the TMPRSS2-ERG fusion gene, which as an oncogene present in approximately 50% of PCas [33] which promotes PCa survival, proliferation and angiogenesis [34-36]. We have found that in serum free medium, which does not contain androgens, FGF23 can induce expression of TMPRSS2 in LNCaP and VCaP cells and the TMPRSS2/ERG fusion gene mRNA and protein in VCAP cells, which contain this gene fusion. The fold induction by FGF23 is less than androgens [33] but may be relevant in men treated with androgen ablation therapies. In this context, androgen receptor activity is extremely low and FGF23 may provide a stimulus for continued ERG expression, particularly in the bone microenvironment, although probably at lower levels than those seen with intact androgen receptor signaling. Further studies are needed to determine the exact interactions between androgen receptor activity, the TMPRSS2/ERG fusion gene and FGF23 signaling.

Aberrant FGF signaling can promote tumor development by directly driving cancer cell proliferation, invasion and survival and by supporting tumor angiogenesis [1-10, 37]. These observations make FGF signaling networks increasingly attractive as targets for therapeutic intervention in PCa. The current studies add further support for this concept. We have recently shown that inhibition of FGFR kinase activity using a small molecule inhibitor markedly decreases PCa tumor progression *in vivo* in a mouse xenograft model [7]. Recent studies by Wan et al [10] have shown that dovatinib, which inhibits FGFR activity (along with several other kinases) inhibits mouse PCa xenograft tumor growth in bone. More importantly, dovitinib treatment of men with advanced metastatic PCa resulted in stable disease or partial or complete responses in the majority of men. These studies provide further support for use of FGFR inhibitors, including highly specific FGFR inhibitors, in men with aggressive PCa.

## MATERIALS AND METHODS

### Cell culture

Human PCa cells PC3, LNCaP, DU145 and 22RV1 were maintained in RPMI-1640 medium (Invitrogen) supplemented with 10% fetal bovine serum (FBS, Invitrogen) and 100 ug/ml penicillin/streptomycin. VCAP and LAPC4 Cell lines were maintained in Dulbecco's Modified Eagle Medium (DMEM, Invitrogen) supplemented with 10% FBS and 1% penicillin/streptomycin (Invitrogen). PC346C cells were cultured in Dulbecco's modified Eagle's medium-Ham's F-12 medium ( Invitrogen) supplemented with 0.1 nm R1881, 2% fetal calf serum (PAN Biotech), 1% insulin-transferrin-selenium (Invitrogen), 0.01% BSA (Roche), 10 ng/ml epidermal growth factor (Sigma-Aldrich), 1% penicillin/streptomycin, 100 ng/ml fibronectin (Harbor Bio-Products), 20 μg/ml fetuin (ICN Biomedicals), 50 ng/ml cholera toxin (Sigma-Aldrich), 0.1 mm phosphoethanolamine (Sigma-Aldrich), and 0.6 ng/ml triiodothyronine (Sigma-Aldrich). All cell lines are authenticated by STR analysis at MD Anderson Cancer Center Characterized Cell Line Core Facility.

### Prostate and prostate cancer tissues

Tissue samples were obtained from the Human Tissue Acquisition and Pathology Core of the Dan L. Duncan Cancer Center and were collected from fresh radical prostatectomy specimens after obtaining informed consent under an Institutional Review Board approved protocol. Cancer samples contained a minimum of 70% cancer and benign tissues were free of cancer on pathological examination. RNA was extracted as described previously [8].

### RT-PCR and quantitative RT-PCR

To examine expression of mRNA in human PCa cell lines and/or patient PCa and benign prostate samples, we carried out RT-PCR and/or quantitative real-time RT-PCR (Q-RT-PCR). Total RNA was extracted using the RNeasy kit (Qiagen). cDNA was synthesized using an iScript cDNA Synthesis kit (BioRad) with a blend of Oligo(dT) and random hexamer primers in a PTC-200 thermocycler (5 min at 25°C; 30 min at 42°C; 5 min at 85°C). Real-time quantitative PCR was performed on the StepOne Plus Real-Time PCR System (Applied Biosystem) using standard parameters. Differences in mRNA levels were analyzed using the 2^−Δ ΔCT^ method. Primer sequences and PCR conditions are summarized in Supplementary Table 2. All data were normalized to HPRT expression. Each measurement point was repeated at least in triplicate and the average and standard deviation were calculated.

### FGF23 enzyme-linked immunoabsorption assay (ELISA)

FGF23 protein expression in PCa cell lines and stable shRNA mediated FGF23 gene knockdown PC3 cells was quantified by ELISA. For cell lysate preparation, cells were grown in growth medium for 72 hour, and cell lysates were prepared. For conditioned medium, cells were seeded in 6-well plates in growth medium and the next day growth medium was removed, cells were washed once with serum-free medium, and cells incubated in serum-free medium for 72 hour. The conditioned media was collected and centrifuged for 10 min at 4000 rpm at 4°C. The protein concentration in cell lysates and conditioned media were determined using BCA protein assay kit (Thermo) and 50 μl of protein lysates were subjected to ELISA assay with a human FGF23 ELISA kit (EMD Millipore) as per the manufacturer's instructions.

### Cell proliferation assays

PCa cells were plated in to 96-well plates at 3×10^3^ cells per well in growth medium. For FGF23 stimulation experiments, cells were then synchronized with serum starvation for 24 hours before the stimulation with FGF23 (R&D Systems) in serum-free medium for 48 hrs. The effect of FGF23 knockdown on the proliferation of PC3 and LNCaP cells was assessed in 96-well plates in complete growth medium. Cell proliferation was determined using the CellTiter 96 Aqueous One Solution Cell Proliferation Assay (Promega) as described by the manufacturer. The absorbance was read at 490 nm with VERSAmax Tunable microplate reader (Conquer Scientific).

### Matrigel invasion assays

Cell invasion assays were performed with BD BioCoat Matrigel invasion chambers (Becton Dickinson). After incubation with 25 ng/mL of FGF23 or vehicle only and 20U/ml of Heparin for 24 hours (PC3) or 48 hours (LNCaP), non-invading cells in the upper chambers were removed and the cells that penetrated through the matrigel to the lower surface of the filter were fixed and stained with Diff-Quik stain. The membranes were mounted on slides and scanned, photographed and all cells were counted. Each treatment was assayed in triplicate and three independent experiments were carried out.

### Soft agar colony formation assay

Cell suspensions of 3000 cells/ml were prepared in 0.35% agar diluted in serum free medium with different doses of FGF23 or vehicle only and 20U/ml Heparin and plated on a 0.6% agar foundation in 6-well culture plates at 37°C. After culture for 14 to 21 days, cells were stained with 2 mg/ml of p-iodonitrotetrazolium violet (Sigma, St Louis, MO) and colonies were counted with a dissecting microscope.

### Western blot

Total cellular protein lysate was prepared as described previously [6]. Briefly, cells were washed once with cold phosphate buffered saline and lysed in modified RIPA buffer containing Tris 50 mM, NaCl 150 mM, Triton X-100 1%, SDS 0.1%, deoxycholate 0.5%, sodium orthovanadate 2 mM, sodium pyrophosphate 1mM, NaF 50mM, EDTA 5 mM, PMSF 1 mM and 1x protease inhibitor cocktail (Roche) and clarified by centrifugation. Protein concentration of the lysates was determined using BCA protein assay kit (Thermo Scientific). 40μg of the extracted protein from each sample was subjected to electrophoresis in 10% sodium dodecyl sulfate (SDS) polyacrylamide gels. Proteins in the gels were transferred onto nitrocellulose membranes (Invitrogen) and subjected to Western blotting with different antibodies. The antibodies (Ab) from Cell Signaling Technology Inc, including phospho-p44/42 MAPK (p-ERK1/2) rabbit mAb; p44/42 (Erk1/2) rabbit mAb; phospho-MEK1/2 (p-MEK1/2) rabbit mAB; MEK1/2 rabbit mAb; phospho-AKT (Ser473) rabbit mAb; total AKT rabbit mAb; and phospho-NF-κB p65 (Ser536) rabbit IgG, were all used at a 1:1000 dilution. Anti-ERG rabbit IgG (Abcam) was used at 1: 2000 dilution. A goat polyclonal anti-β-actin antibody (Santa Cruz Biotech) was utilized at 1:5000 as loading control. After incubation with primary antibodies overnight at 4°C or for 1 hour at room temperature, horseradish peroxidase-labeled secondary antibodies were then applied to the membranes for 1 h at room temperature. Signals were visualized using enhanced chemiluminescence Western blotting detection reagents (Thermo).

### Stable knockdown of FGF23

PC3 and LNCaP cells were plated at a density of 5 × 10^5^ cells/well in 6-well plates, in antibiotic-free growth medium. The next day cells were transfected with 2 ug of shRNA plasmid (pGIPZ vector) or shFGF23 plasmid (V3LHS-59221, shFGF23-I and V3LHS-305090, shFGF23-II; GE Healthcare Dharmacon) with FuGENE 6 Transfection Reagent (Roche) according to the manufacture's instruction. Puromycin (1ug/ml) was added three days later for selection of transfected cells. Stably transfected cells were then maintained in RPMI growth medium supplemented with puromycin.

### Mouse xenograft experiments

All experiments were carried out on 8- to 12 week-old male SCID mice in accordance with the IACUC approved protocol. Tumor xenografts were established by subcutaneous injection over each flank with either PC3 cells with stable FGF23 knockdown or vector controls cells (3 × 10^6^ cells/site) mixed 1:1 with Matrigel (Becton Dickinson). The tumor size was measured twice weekly using calipers and the tumor volume in mm3 calculated as volume = (height × width × length)/2. Tumors were harvested 42 days after the cell inoculation and tumors were excised and weighed.

### Expression array analysis

LNCaP cells were plated at 6-well plates and left to grow for 24 hours. Cells were serum-starved for 24 hours and then stimulated with 25 ng/ml of FGF2, FGF19 or FGF23 (all from Becton Dickinson) for 24 hours. Total RNAs were extracted with mini RNeasy kit as described above. RNAs were labeled and hybridized to Agilent 60K arrays as described previously [6]. Array data have been deposited into the Gene Expression Omnibus (GSE62192). For each treatment group, top differentially expressed genes relative to control were defined (using fold change >1.4 for each FGF profile compared to each control profile), and the set of top differential genes found for any treatment group were clustered, using a supervised approach as described elsewhere [38]. Expression patterns were visualized as color maps using Java TreeView [39]. VCaP cells were treated similarly for analysis of TMPRSS2 and TMPRSS2/ERG expression. Primers for TMPRSS2/ERG and Western blot for ERG and phospho NF-kB p65-Ser536 were performed as described previously [20, 34, 35].

### Statistical analysis

Numerical values were compared using t-test (two sided). Differences were considered significant if p<.05.

## SUPPLEMENTARY MATERIAL FIGURE AND TABLES




