# Supplementary material for: FGF23 promotes prostate cancer progression
Source: Oncotarget. 2015 May 19;6(19):17291–301. doi: 10.18632/oncotarget.4174 (PMC4627308; doi:10.18632/oncotarget.4174)
Supplement: Supplementary file 1 [file oncotarget-06-17291-s001.pdf]

FGF23 promotes prostate cancer progression

Supplementary Material

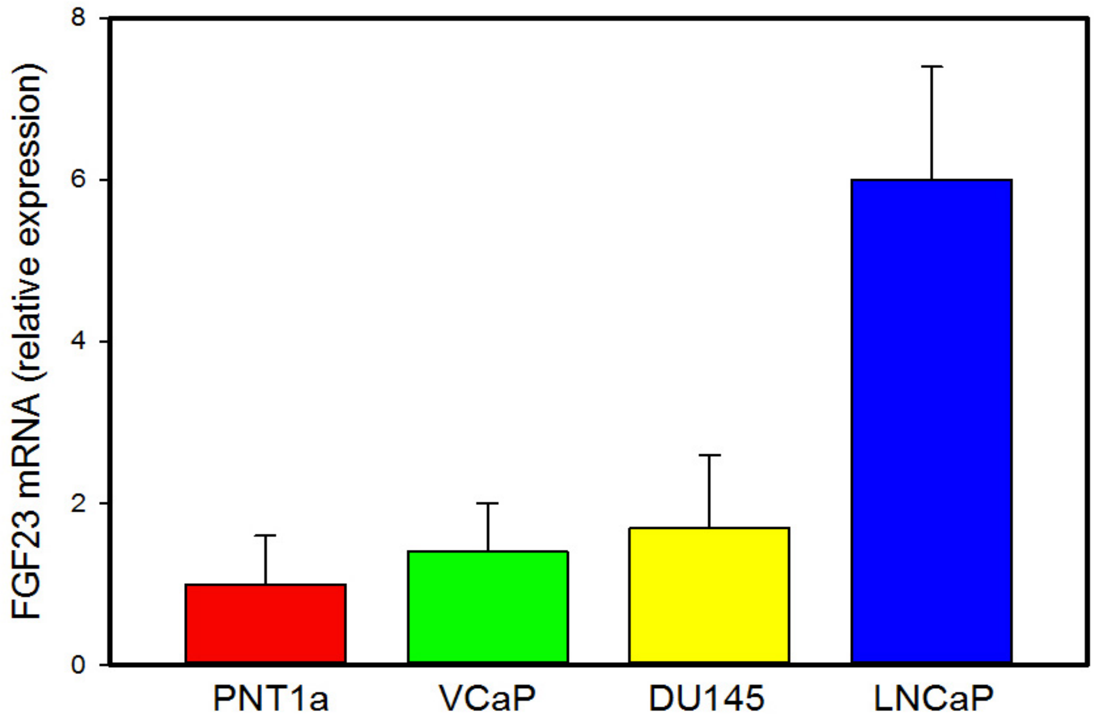

Supplementary Table 2. Primers used for conventional RT-PCR and real-time quantitative RT-PCR

Gene	Primer sequence	Amplicon size (bp)
FGF23	FGF23-E1F: 5-CACAGCCACAGCCAGGAACAGC-3<br>FGF23E3R: 5-GTCGTACCCGTTTTCCAGCGTCT-3	245
KL	KL-F: 5-GGGACCACCAAGAGAGATGA-3<br>KL-R: 5'-TCCCTTCTAGGGCTGATTT-3'	292
VEGFA	VEGFA-F 5-CAAGACAAGAAAATCCCTGTGG-3<br>VEGFA-R 5-GCTTGTCACATCTGCAAGTACG-3	156
TMPRSS2	TMPRSS2-F: 5-CCTGCATCAACCCCTCTAACTG-3<br>TMPRSS2-R: 5-AGGCGAACACACCGATTCTC-3	79
PKIB	PKIB-F 5-GATGGGCAAATCATTCTTGGTA-3<br>PKIB-R: 5-GGCACATACTAGAAGCAAAATACG-3	261
ABCC4	MRP4-F: 5-GTTCTTCTGGTGGCTCAATCC-3<br>MRP4-R: 5-GGCTTCTGTGCGTCATTCTC-3	168
TXNIP	TXNIP-F: 5- ACTCGTGTCAAAGCCGTTAGG-3<br>TXNIP-R: 5'- TCCCTGCATCCAAAGCACTT-3'	63
EGR3	EGR3-F: 5-TCACCACTCACATCCGCACTCATA-3<br>Egr3-R: 5-TTTGCTTGAGGTGGATCTTGGCGT-3	117
HPRT	HPRT-F: 5-GCAGACTTTGCTTTCCTTGG-3<br>HPRT-R: 5-TCAGGGATTTGAATCATGTTTG-3	189
